# Supplementary material for: On-surface synthesis of tailored organic platforms for single metal atoms
Source: Nat Commun. 2025 Dec 3;16:10597. doi: 10.1038/s41467-025-66171-3 (PMC12675580; doi:10.1038/s41467-025-66171-3)
Supplement: Supplementary file 1 — Supplementary Information [file 41467_2025_66171_MOESM1_ESM.pdf]

Supplementary Information

for

On-Surface Synthesis of Tailored Organic Platforms for Single Metal Atoms

Amogh Kinikar,[1]§ Xiushang Xu,[2]§ Takatsugu Onishi,[2] Andres Ortega-Guerrero,[1] Roland Widmer,[1] Nicola Zema,[3] Conor Hogan,[3] Luca Camilli,[4] Luca Persichetti,[4] Carlo A. Pignedoli,[1] Roman Fasel,[1,5] Akimitsu Narita,[2]* Marco Di Giovannantonio[3]*

[1]*Empa, Swiss Federal Laboratories for Materials Science and Technology, nanotech@surfaces Laboratory, 8600 Dübendorf, Switzerland*
[2]*Organic and Carbon Nanomaterials Unit, Okinawa Institute of Science and Technology Graduate University, 904-0495 Okinawa, Japan*
[3]*CNR – Istituto di Struttura della Materia (CNR-ISM), 00133 Roma, Italy*
[4]*Dipartimento di Fisica, Università di Roma "Tor Vergata", 00133 Roma, Italy*
[5]*Department of Chemistry, Biochemistry and Pharmaceutical Sciences, University of Bern, Bern 3012, Switzerland*
§*These authors contributed equally to this work*

*Corresponding authors: Akimitsu Narita (akimitsu.narita@oist.jp), Marco Di Giovannantonio (marco.digiovannantonio@cnr.it).

Content:

Complete synthetic procedure for DBAP-tpy
Additional experimental and computational results
CO_2 behavior vs T
Control experiments
NMR spectra
References

Complete synthetic procedure for DBAP-tpy

DBAP-tpy (**1**) was synthesized as shown below. Diiodobenzene **2** was lithiated and reacted with aldehyde **3**, which was synthesized by palladium-catalyzed C(sp3)-H arylation of 5-bromo-2-methylbenzaldehyde with 1-bromo-4-iodobenzene[1,2]. Subsequently, the obtained hydroxy intermediate was treated with BF_3•OEt_2 and oxidized by 2,3-dichloro-5,6-dicyano-1,4-benzoquinone (DDQ) to afford 2,7-dibromo-9-(4-iodophenyl)anthracene (**4**) in 46% yield. Then, **4** was subjected to regioselective halogen-metal exchange reaction with isopropylmagnesium chloride at –78 °C, and reacted with dimethylformamide (DMF) to produce aldehyde **5** in 68% yield. Finally, DBAP-tpy **1** was obtained from **5** and 2-acetylpyridine[3] in 45% yield.

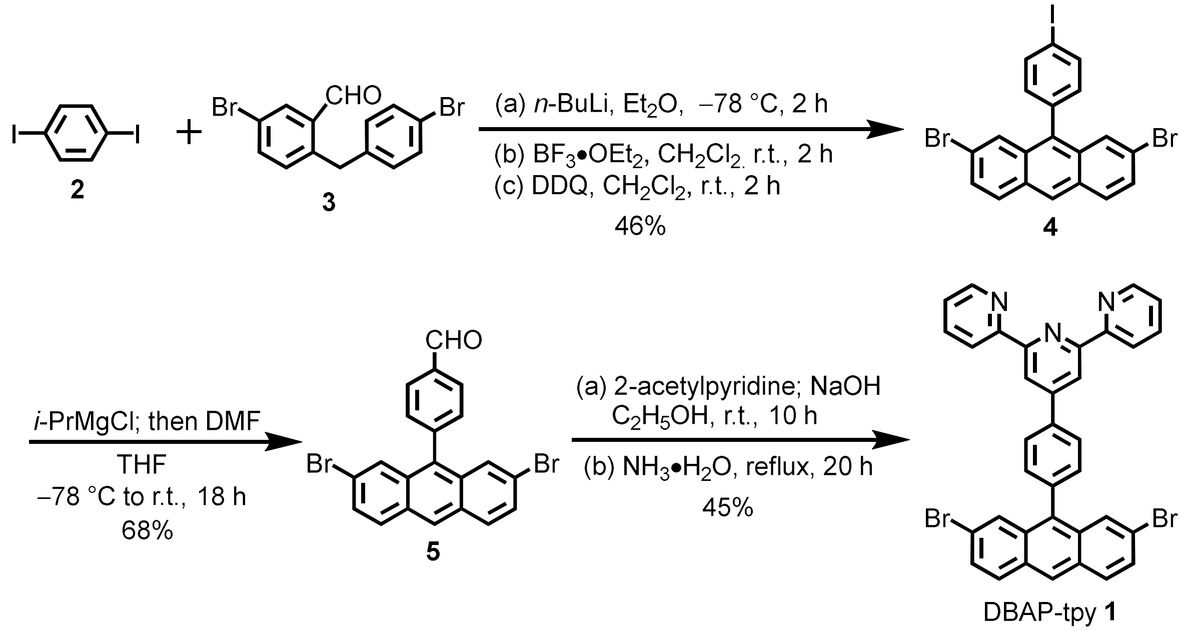

2,7-dibromo-9-(4-iodophenyl)anthracene (**4**)

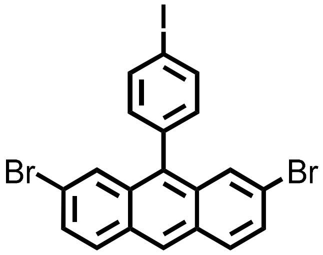

To a solution of 1,4-diiodobenzene (**2**) (100 mg, 0.303 mmol) in anhydrous tetrahydrofuran (THF) (10.0 mL), *n*-butyllithium (1.6 M in hexane, 0.20 mL, 0.32 mmol) was added dropwise at –78 °C under argon atmosphere. After reaction mixture was stirred for 2 h, 5-bromo-2-(4-bromobenzyl)benzaldehyde (**3**) (106 mg, 0.303 mmol) was added. Then, the resulting mixture was stirred overnight at room temperature. The reaction was quenched with saturated aqueous solution of NH_4Cl (10 mL) and extracted with CH_2Cl_2 (10 mL) for three times. The organic phases were combined, washed with brine, dried with $MgSO_4$, and evaporated. The residue was subsequently dissolved in dry CH_2Cl_2 (10 mL), and BF_3•OEt_2 (0.05 mL, 0.4 mmol) was added at 0 °C under argon atmosphere. Then, the reaction mixture was stirred 2 h at 0 °C. The resulting mixture was quenched with saturated aqueous solution of $NaHCO_3$ (10 mL) and extracted with CH_2Cl_2 (10 mL) for three times. The organic phases were combined, washed with brine, dried with $MgSO_4$, and evaporated. The residue was then dissolved in CH_2Cl_2 (30 mL) and DDQ (209 mg, 0.909 mmol) was added to the solution, followed by stirring at room temperature for 2 h. The reaction mixture was poured into water (10 mL) and extracted with CH_2Cl_2 (10 mL) for three times.

Then, organic phases were combined, washed with brine, dried over $MgSO_4$, and evaporated. The residue was purified by silica gel column chromatography (eluent: hexane) to give the title compound (70 mg, 46% yield) as light yellow solid. ^{1}H NMR (500 MHz, $CDCl_3$) δ 8.43 (s, 1H), 7.95 (d, J = 7.9 Hz, 2H), 7.90 (d, J = 9.0 Hz, 2H), 7.75 (d, J = 1.8 Hz, 2H), 7.54 (dd, J = 9.0, 1.9 Hz, 2H), 7.13 (d, J = 7.9 Hz, 2H). ^{13}C NMR (126 MHz, $CDCl_3$) δ 137.00, 135.84, 132.95, 131.94, 130.24, 129.15, 128.55, 128.23, 127.21, 126.48, 119.95. HRMS (MALDI-TOF, Positive): *m/z* Calcd. For $C_{20}H_{11}Br_2I^+$: 535.8672 $[M]^+$, found: 535.8696.

4-(2,7-dibromoanthracen-9-yl)benzaldehyde (**5**)

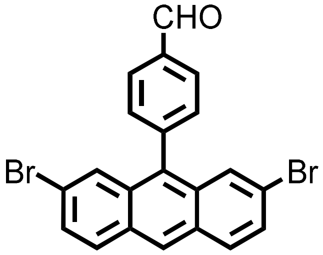

To a solution of compound **4** (570 mg, 1.06 mmol) in anhydrous THF (30 mL) was added *i*-PrMgCl (2.0 M in THF, 0.5 mL, 1.0 mmol) dropwise at –78 °C under argon atmosphere. After stirring the reaction mixture at –78 °C for 2 h, dimethylformamide (DMF) (155 mg, 2.12 mmol) was added. The reaction mixture was allowed to warm to room temperature and stirred overnight at room temperature for 18 h. The reaction was quenched by saturated aqueous solution of $NaHCO_3$ (20 mL) and extracted with CH_2Cl_2 (30 mL) for three times. The organic layers were combined, washed with brine, dried with $MgSO_4$, and evaporated. The residue was purified by silica gel column chromatography (eluent: hexane: ethyl acetate = 20:1) to afford the title compound (320 mg, 68%) as yellow solid. ^{1}H NMR (400 MHz, $CDCl_3$) δ 10.22 (s, 1H), 8.65 – 8.32 (m, 1H), 8.15 (t, J = 6.0 Hz, 2H), 7.99 – 7.86 (m, 2H), 7.76 – 7.63 (m, 2H), 7.65 – 7.45 (m, 4H). ^{13}C NMR (101 MHz, $CDCl_3$) δ 191.90, 143.99, 140.72, 136.49, 136.10, 133.85, 131.94, 131.47, 131.04, 130.16, 129.55, 129.34, 127.99, 127.87, 126.73, 125.35, 122.24, 121.20. MS (MALDI-TOF, Positive): *m/z* Calcd. For $C_{20}H_{12}Br_2O^+$: 434.92 $[M]^+$, found: 434.92.

4'-{4-(2,7-dibromoanthracen-9-yl)phenyl}-2,2':6',2''-terpyridine (DBAP-tpy **1**)

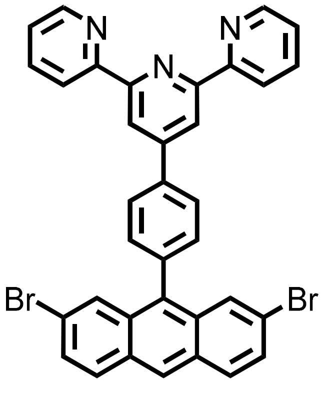

To a solution of NaOH (106 mg, 2.63 mmol) in 10 mL of C_2H_5OH, compound **5** (210 mg, 0.480 mmol) and 2-acetylpyridine (128 mg, 1.05 mmol) were added. After stirring at room temperature for 10 h, aqueous NH_3•H_2O (3.0 mL) was added and the mixture was refluxed for 20 h. After cooling to room temperature, ethanol was removed in vacuo. The aqueous phase was extracted with CH_2Cl_2 (30 mL) for three times. The organic layers were combined, washed with brine, dried with $MgSO_4$, and evaporated. The residue was purified by silica gel column chromatography (eluent: CH_2Cl_2: methanol = 200:1) to afford the title compound (138 mg, 45%) as light yellow solid. ^{1}H NMR (400 MHz, $CDCl_3$) δ 8.92 (s,

2H), 8.77 (dd, $J = 4.8$, 1.9 Hz, 2H), 8.73 (dt, $J = 8.0$, 2H), 8.45 (s, 1H), 8.19 – 8.08 (m, 2H), 7.94 – 7.89 (m, 4H), 7.87 – 7.81 (m, 2H), 7.55 (dd, $J = 8.7$, 1.6 Hz, 4H), 7.40 – 7.37 (m, 2H), 7.26 (s, 2H). ^{13}C NMR (101 MHz, $CDCl_3$) δ 156.08, 149.91, 149.20, 138.49, 138.00, 136.89, 134.92, 131.68, 131.41, 130.12, 129.60, 129.20, 128.49, 127.81, 127.28, 123.90, 121.36, 120.86, 119.08. HRMS (ESI, Positive): *m/z* Calcd. For $C_{35}H_{22}Br_2N_3$: 642.0143 $[M+H]^+$, found: 642.0143.

Additional experimental and computational results

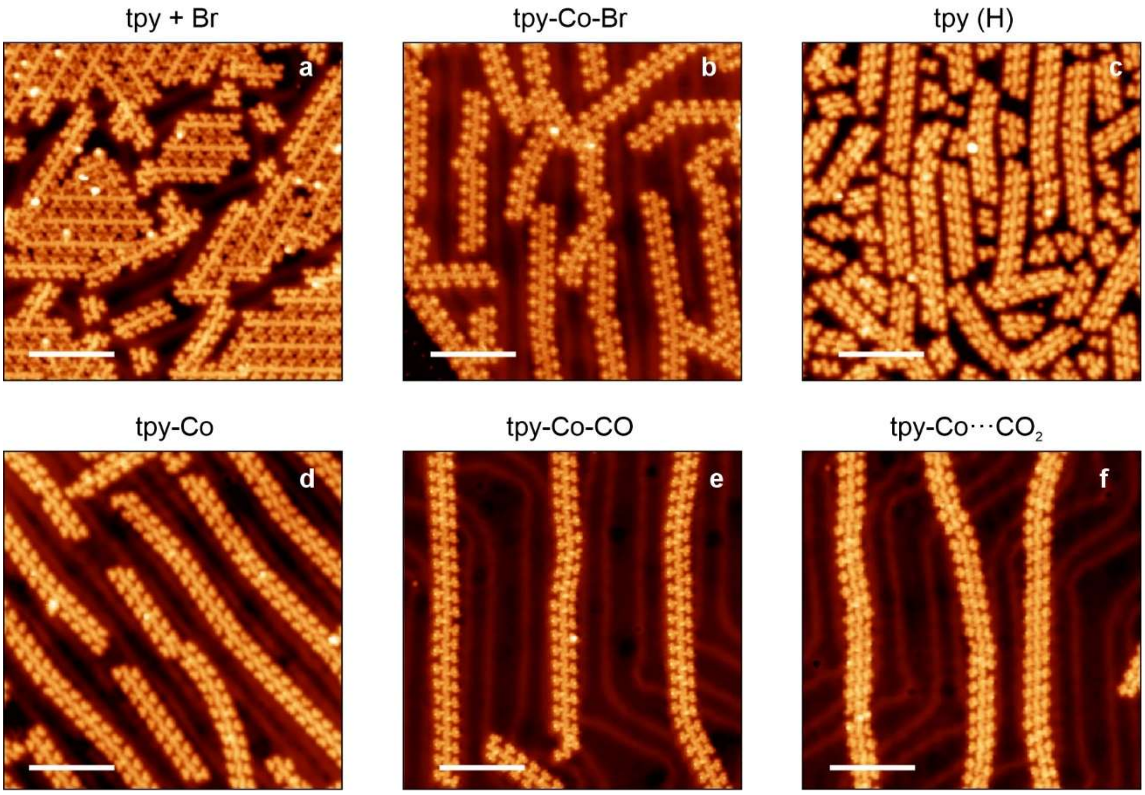

Supplementary Fig. 1 | Large scale STM images. STM images of the samples after different preparation processes. **a,** After the growth of polymer chains. Bromine atoms detached from the precursor molecules during the dehalogenative aryl-aryl coupling chemisorb on the surface and are visible in between the polymers. **b,** After dosing cobalt on a sample grown as in **a**. Tpy-Co-Br complexes are formed. **c,** After dosing atomic hydrogen on a sample grown as in **a**. Bromine is no longer visible on the surface. The polymer chains are not packed in islands anymore, but appear well separated from each other. **d,** After dosing cobalt on a sample grown as in **c**. Although not evident from large scale STM images, all tpy sites feature coordinated cobalt atoms (tpy-Co). **e,** After dosing CO on a sample grown as in **d**, and subsequently annealed on the wobblestick for 15 minutes. Nearly all units consist of CO bound to the active site (tpy-Co-CO). **f,** After dosing CO_2 on a sample grown as in **d**, and subsequently annealed on the wobblestick for 2 minutes. Most of the units feature CO_2 molecules sitting next to the active site (tpy-Co···CO_2). Scanning parameters: $I_t = 70$ pA, $V_b = -0.50$ V (**a**); $I_t = 50$ pA, $V_b = -0.20$ V (**b**); $I_t = 70$ pA, $V_b = -0.50$ V (**c**); $I_t = 200$ pA, $V_b = -0.02$ V (**d**); $I_t = 70$ pA, $V_b = -0.15$ V (**e**); $I_t = 100$ pA, $V_b = -0.10$ V (**f**). Scale bars: 10 nm (**a-f**).

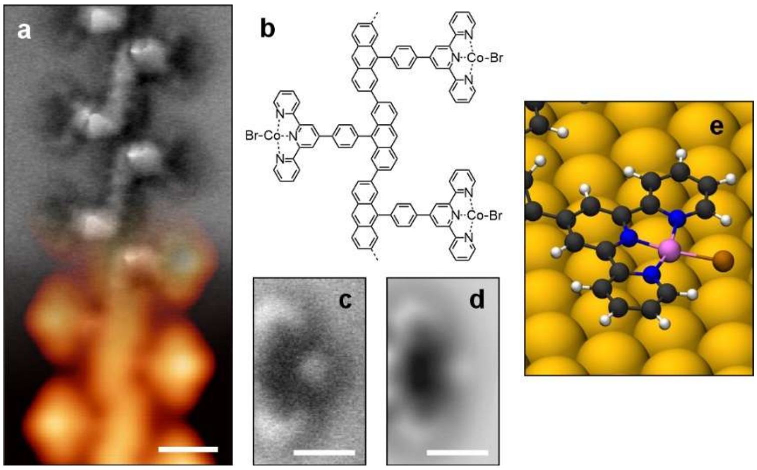

Supplementary Fig. 2 | Tpy-Co-Br units. a, Experimental STM (I_t = 100 pA, V_b = –0.1 V) and nc-AFM (Δz = +210 pm) images of a polymer segment where the tpy-Co sites are passivated by bromine atoms (tpy-Co-Br). **b,** Chemical scheme of the obtained polymer. **c,** Zoom-in experimental nc-AFM image (Δz = +200 pm) of a tpy-Co-Br unit. **d,** Simulated nc-AFM image of tpy-Co-Br unit obtained from the structure in **e**. **e,** Zoom-in of the DFT-optimized geometry of a polymer segment with tpy-Co-Br units on Au(111). Scale bars: 1 nm (**a**), 0.5 nm (**c,d**).

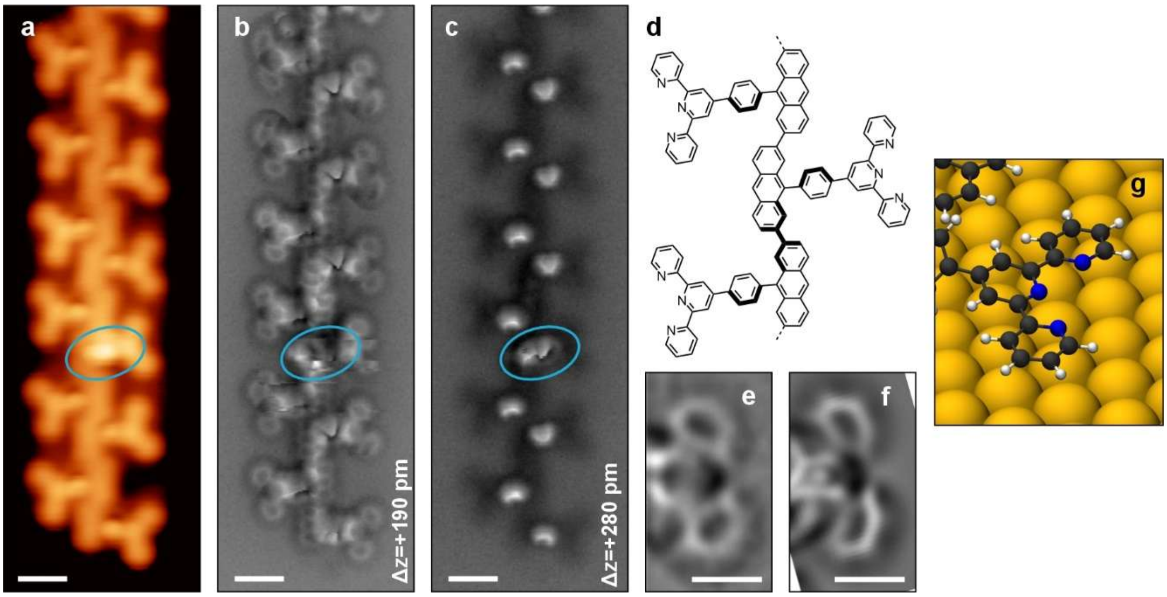

Supplementary Fig. 3 | Polymer chains after exposure to atomic hydrogen. a-c, Experimental STM (**a**, I_t = 50 pA, V_b = –0.5 V) and nc-AFM (**b,c**) images of a polymer segment after exposure to atomic hydrogen. Some parts of the polymer backbone present defects (cyan ellipse in **a-c**). These defects appear as bright protrusions in the STM and nc-AFM images and their unambiguous identification is not trivial. We speculate that they can be due to the hydrogenation of some parts of the chain, which however does not affect the structure of the tpy units. **d,** Chemical scheme of the obtained polymer. **e,** Zoom-in experimental nc-AFM image (Δz = +220 pm) of a tpy unit. **f,** Simulated nc-AFM image of tpy unit obtained from the structure in **g**. **g,** Zoom-in of the DFT-optimized geometry of a polymer segment on Au(111) with tpy units where the nitrogen atoms are all pointing towards the center of the tpy. Scale bars: 1 nm (**a-c**), 0.5 nm (**e,f**).

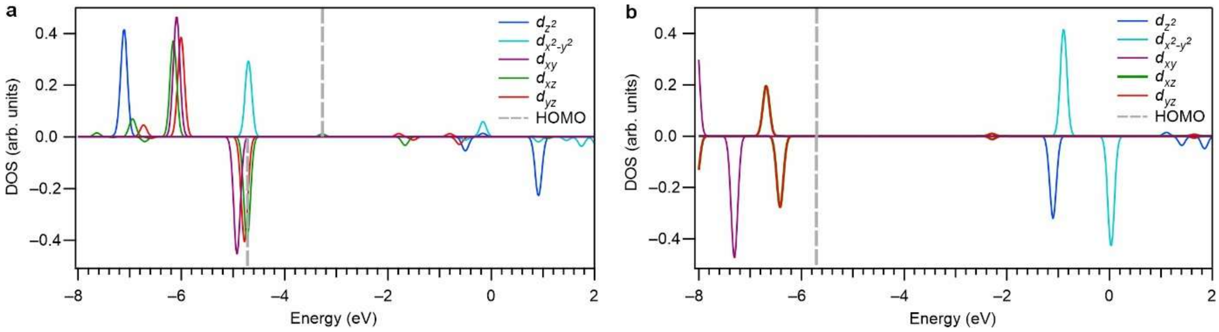

Supplementary Fig. 4 | PDOS of Co 3*d* orbitals. a. PDOS of the 3*d* orbitals of cobalt in the tpy-Co compound. **b.** PDOS of the 3*d* orbitals of cobalt in the Co-porphyrin. The graphs elucidate the origin of the spectral features reported in Fig. 3c,f in the main text. Positive (negative) values in the y-axis correspond to the spin-up (spin-down) channel. Highest occupied molecular orbital (HOMO) levels are indicated by the grey dashed lines.

Supplementary Table 1 | Summary of the Natural Population Analysis (NPA) from the Natural Bond Orbital (NBO) Analysis of the Co atom in tpy-Co and Co-porphyrin.

Natural electron configuration Co atom	tpy-Co	Co-porphyrin
Alpha	[core]4s(0.19)3d(4.84)4p(0.18)	[core]4s(0.14)3d(4.30)4p(0.20)
Beta	[core]4s(0.07)3d(3.03)4p(0.08)	[core]4s(0.10)3d(3.29)4p(0.19)
Total	[core]4s(0.26)3d(7.86)4p(0.26)4d(0.01)	[core]4s(0.25)3d(7.59)4p(0.38)4d(0.01)

The results are presented as the effective valence electron configuration, also referred to as the *Natural Electron Configuration*. The analysis is provided separately for the α and β spin densities, as well as for the overall total. The reported values show a higher *d* orbital occupancy for the tpy-Co.

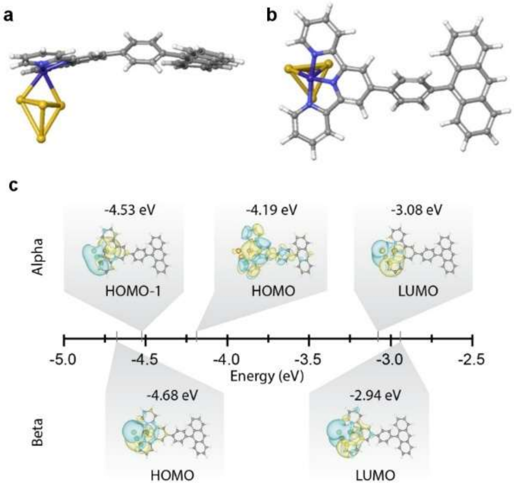

Supplementary Fig. 5 | Molecular representation of the tpy-Co with a Au_4 cluster. The molecular structure and the gold cluster are extracted from the DFT-optimized geometry of the tpy-Co on a Au(111) surface, and the atomic positions are constrained. Side view (**a**) and top view (**b**) are reported. (**c**) Gas phase DFT molecular orbital diagram of the frontier orbitals for the system in panels a, b. The molecule is in a doublet spin state and both alpha and beta spin states are reported, highlighting the broken symmetry of the states in the active site.

Supplementary Table 2 | DFT Calculated Energy of tpy-Co with the Au_4 Cluster Model.

System	Energy[eV]
Tpy-Co ($s=\frac{1}{2}$)+ Au_4 cluster	-93502.972
Tpy-Co ($s=\frac{3}{2}$)+ Au_4 cluster	-93502.663
$\Delta E\ [s=\frac{1}{2} - s=\frac{3}{2}]$	-0.309

The table presents the DFT calculated single point energies for the tpy-Co complex with the Au_4 cluster model. These geometries were derived from a representative model built using periodic calculations (tpy-Co on the Au(111) surface). In this model, both possible spin configurations for Co were considered. Among these, the doublet spin configuration was found to be the most energetically favorable.

Supplementary Table 3 | DFT Calculated Energies of tpy-Co in Doublet and Quartet Spin Configurations.

System	Energy[eV]
Tpy-Co ($s=\frac{1}{2}$)	-78727.168
Tpy-Co ($s=\frac{3}{2}$)	-78727.726
$\Delta E\ [s=\frac{1}{2} - s=\frac{3}{2}]$	0.558

The table presents the DFT calculated energies for the tpy-Co complex in both doublet and quartet spin states in the gas phase. The structural coordinates for both spin states were optimized using the PBE0 exchange-correlation functional. The results indicate that in the gas phase, the quartet spin configuration is energetically more favorable than the doublet spin configuration.

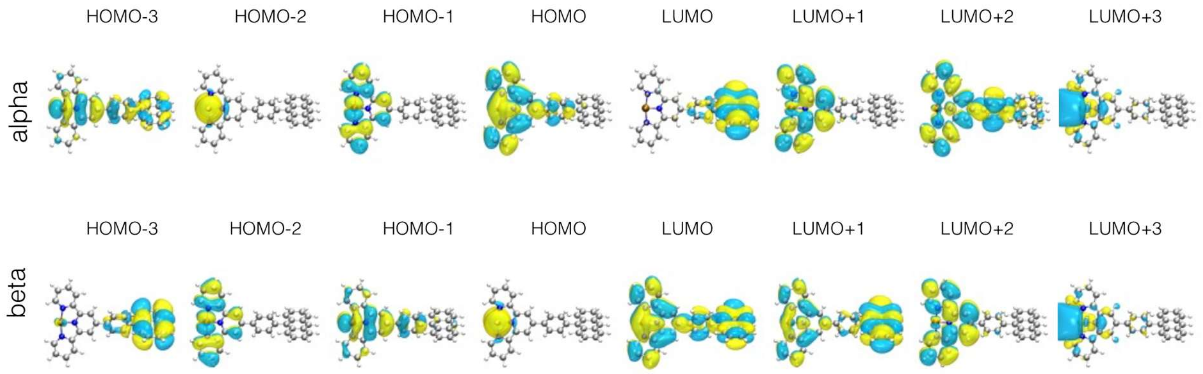

Supplementary Fig. 6 | Simulated Orbitals of tpy-Co in a doublet spin configuration in gas phase. The orbitals of tpy-Co complex are obtained from a geometry optimization calculation in gas-phase, where the multiplicity is set as a doublet.

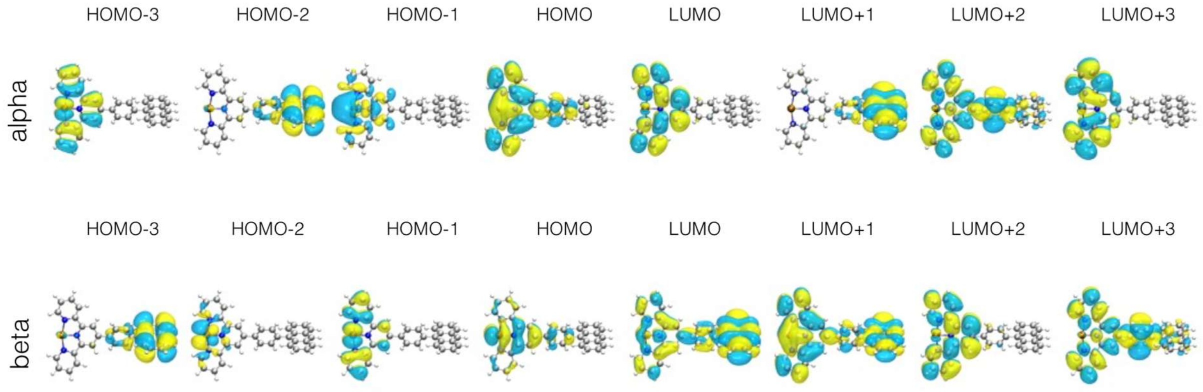

Supplementary Fig. 7 | Simulated Orbitals of tpy-Co in a quartet spin configuration in gas phase. The orbitals of tpy-Co complex are obtained from a geometry optimization calculation in gas-phase, where the multiplicity is set as a quartet.

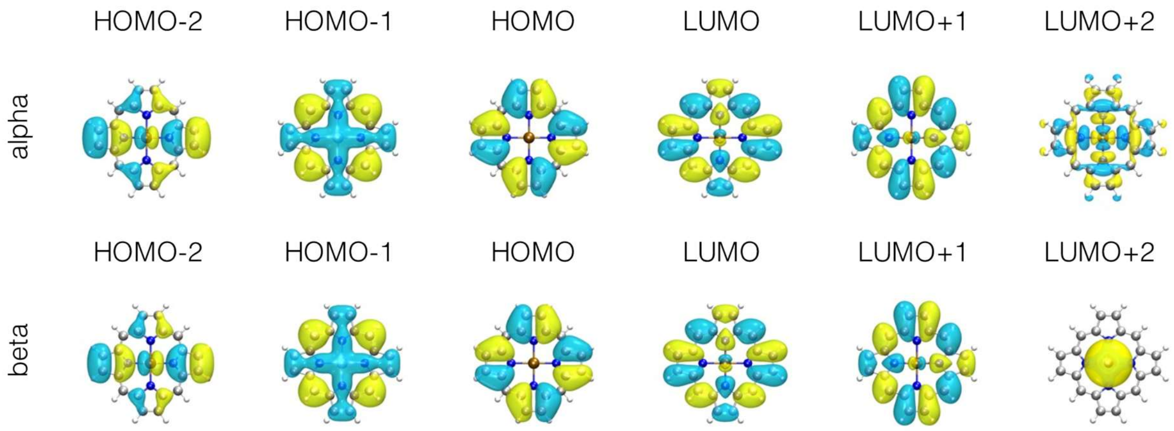

Supplementary Fig. 8 | Simulated orbitals of Co-porphyrin. The diagram illustrates the frontier molecular orbitals of a Co-porphyrin complex, computed using the PBE0 functional with the def2-TZVP basis set. The displayed orbitals demonstrate the characteristic features of the Gouterman model, highlighting the four key π and π^* orbitals. Notably, the diagram shows that the unoccupied orbitals include a dz^2 orbital that is higher in energy relative to the π^* orbitals.

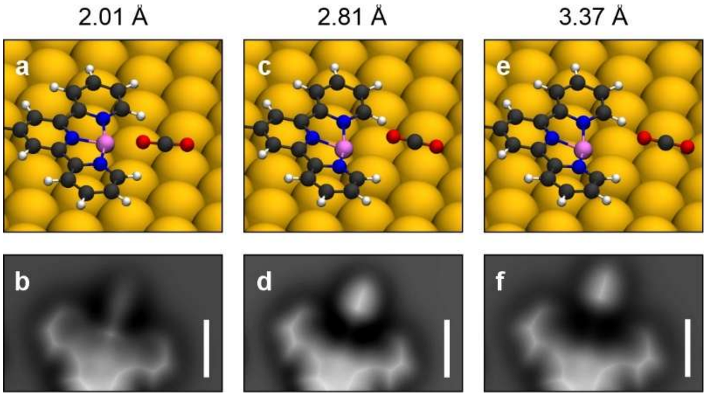

Supplementary Fig. 9 | Identification of the tpy-Co···CO_2 phase. a,c,e, DFT-optimized geometries with the CO_2 molecule at varying distances from the cobalt atom (values on top of the panels indicate the distance between cobalt and the nearest oxygen). The three geometries are obtained after relaxation of different initial guesses. **b,d,f,** Simulated nc-AFM images obtained from the geometries in panels **a**, **c**, and **e**, respectively. The best match with the experimental nc-AFM images in Fig. 5 in the main text is found for an intermediate distance between cobalt and the CO_2 of 2.81 Å. Shorter distance (2.01 Å) leads to an increased interaction between the CO_2 and the cobalt, with the latter being pulled up and becoming visible as a bright dot in the nc-AFM simulation (**b**). Larger distance (3.37 Å) produces a nc-AFM image with the linear feature due to the CO_2 that is too far from the tpy core as compared to the experimentally observed configuration. All these structures are energetically very similar, with their total energy values falling within 0.017 eV. The structure in a,b could represent the experimentally observed specie after annealing of the CO_2 dosed sample, where an increased interaction between CO_2 and Co is promoted (tpy-Co-CO_2). Scale bars: 0.5 nm.

CO_2 behavior vs T

In the captivating encounter between our SAP and CO_2, not only did we witness the presence of individual gas molecules positioned on the surface and in proximity to active sites but also observed them diminishing in favor of different species during stepwise annealing experiments.

A sample exposed to CO_2 while being held at 9.5 K reveals 54% of tpy-Co···CO_2 sites along with 9% of tpy-Co-CO ones. Successive WA steps (see Methods) at increasingly longer times promoted changes in the active site composition (Supplementary Fig. 10 and Supplementary Table 4). The relative abundance of tpy-Co···CO_2 sites diminished in favor of different species, which could be attributed to either tpy-Co-CO or tpy-Co-CO_2 sites. These new species reached a maximum of 63% after 7 minutes of WA and dropped to 8% after 30 minutes, as the sample likely reached a temperature high enough to promote a significant gas desorption. Remarkably, a further WA of 3 minutes (last point in Supplementary Fig. 10) does not lead to any increase of these sites.

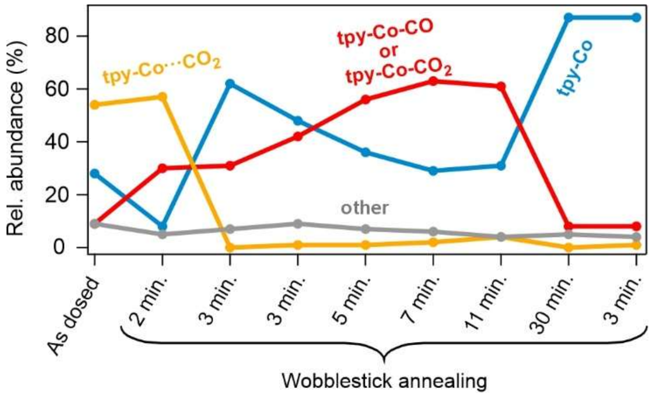

Supplementary Fig. 10 | Evolution of the active sites' occupation. Relative abundance of the tpy-Co sites with different occupation as a function of the residence time of the sample on the wobblestick in consecutive WA steps (see Methods). Data are plotted from the values in Supplementary Table 4.

Supplementary Table 4 | Statistical analysis of the active site composition in CO_2 experiments.

Active site composition	As dosed	WA 2 min.	WA 3 min.	WA 3 min.	WA 5 min.	WA 7 min.	WA 11 min.	WA 30 min.	WA 3 min.
tpy-Co···CO_2	54%	57%	0%	1%	1%	2%	4%	0%	1%
tpy-Co-CO or tpy-Co-CO_2	9%	30%	31%	42%	56%	63%	61%	8%	8%
tpy-Co	28%	8%	62%	48%	36%	29%	31%	87%	87%
others	9%	5%	7%	9%	7%	6%	4%	5%	4%

Relative abundance of the main species as a function of the sample preparation and treatment. The initial sample was obtained by exposing the SAP to CO_2 at 5×10^{-8} mbar for 15 minutes on the SAP-decorated sample at 9.5 K. Subsequent wobblestick annealing (WA) steps were performed, holding the sample on the wobblestick pincer for increasingly longer times. Some features at the active sites could not be assigned clearly and were categorized as "others". The data in this table – used to produce the graph in Supplementary Fig. 10 – were obtained by analyzing several STM images (50×50 nm^2) and counting between 500 and 1000 active sites at each step.

The exact composition of the new sites is crucial, as in one case (tpy-Co-CO) it would reflect an unexpected CO_2-to-CO conversion, while in the other case (tpy-Co-CO_2) a rearrangement of CO_2 in the active sites. The ultimate confirmation of the gas composition during the stepwise annealing, however, requires the investigation of the system with spectroscopic techniques that are complementary to SPMs, and is out of the scope of the present study. For completeness, we report below a series of control experiments that we carried out to confirm that there was no other source of CO during the annealing steps.

Control experiments

1. ***Blank***
   A sample featuring tpy-Co sites was inserted in the cold STM stage, let cool down, and then the same processing as in real CO_2 exposures was performed, but without introducing any CO_2 gas in the chamber. Specifically, when the sample reached the base temperature of 4.7 K, we opened the cryostat shields, waited 15 minutes, and then closed the shields. STM investigation of the resulting sample revealed the complete absence of tpy-Co-CO and tpy-Co···CO_2 sites. This result should be compared to the "As dosed" sample in Supplementary Table 4, where 54% of the tpy-Co sites were occupied by CO_2 and 9% were in the form of tpy-Co-CO/tpy-Co-CO_2.

2. ***Wobblestick annealing of 3 minutes***
   The sample obtained from the previous treatment (blank experiment) was extracted from the cold STM stage, let warm up on the wobblestick for 3 minutes, and inserted again in the STM stage. STM investigation of the resulting sample revealed 1% of tpy-Co-CO/tpy-Co-CO_2 and 5% of tpy-Co···CO_2 sites. These values are within the experimental error of the yield determination analysis, and are considered as negligible. Moreover, some other impurities show sometimes a similar appearance of the tpy-Co···CO_2 sites, and could be confused, justifying the overestimated value of 5%. In particular, the same sample treatment after dosing CO_2 on the sample led to 31% of tpy-Co-CO/tpy-Co-CO_2 sites.

3. ***Argon dosing + wobblestick annealing of 11 minutes***
   Here, we excluded that some CO molecules could reach the surface of the sample after being displaced from the cryostat walls and STM stage due to CO_2 collisions with these surfaces. To do so, we dosed another gas, namely argon, with the same exposure used during the CO_2 experiments. After such Ar dosing on a standard tpy-Co sample, we performed a WA for 11 minutes, which is expected to maximize the abundance of tpy-Co-CO/tpy-Co-CO_2 sites (61% in the experiment with CO_2, see Supplementary Table 4). However, we only found 4% of tpy-Co-CO/tpy-Co-CO_2 sites.

4. ***Ion gauge off***
   In some cases, ionization processes occurring in the proximity of ion gauge filaments (used to monitor the pressure in UHV systems) can induce chemical reactions in the molecules composing the rest gas of the vacuum setup. Here, we excluded that the CO was produced by the ionization of CO_2 from such filament, repeating the experiment with the ion gauge switched off. After cooling down the sample with tpy-Co sites in the STM stage we exposed it to CO_2 as in previous experiments, but keeping the ion gauge off. After the exposure, we performed a wobblestick annealing of 11 minutes. As expected, the tpy-Co-CO/tpy-Co-CO_2 sites amounted to 83%. The significantly higher value as compared to the 61% reported in Supplementary Table 4 could be due to a slightly higher CO_2 partial pressure during the exposure, which could not be kept entirely constant in absence of any pressure readout.

NMR spectra

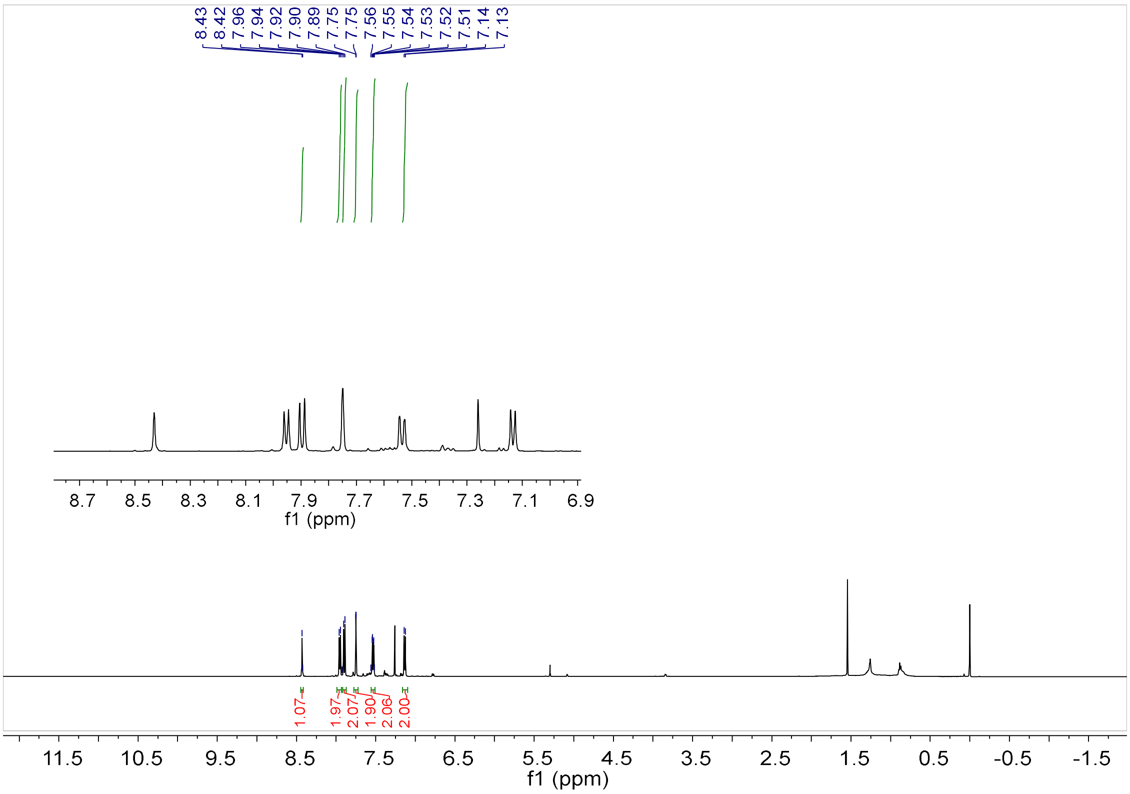

Supplementary Fig. 11 | ^{1}H NMR spectrum of compound **4** in $CDCl_3$ (500 MHz, 298 K).

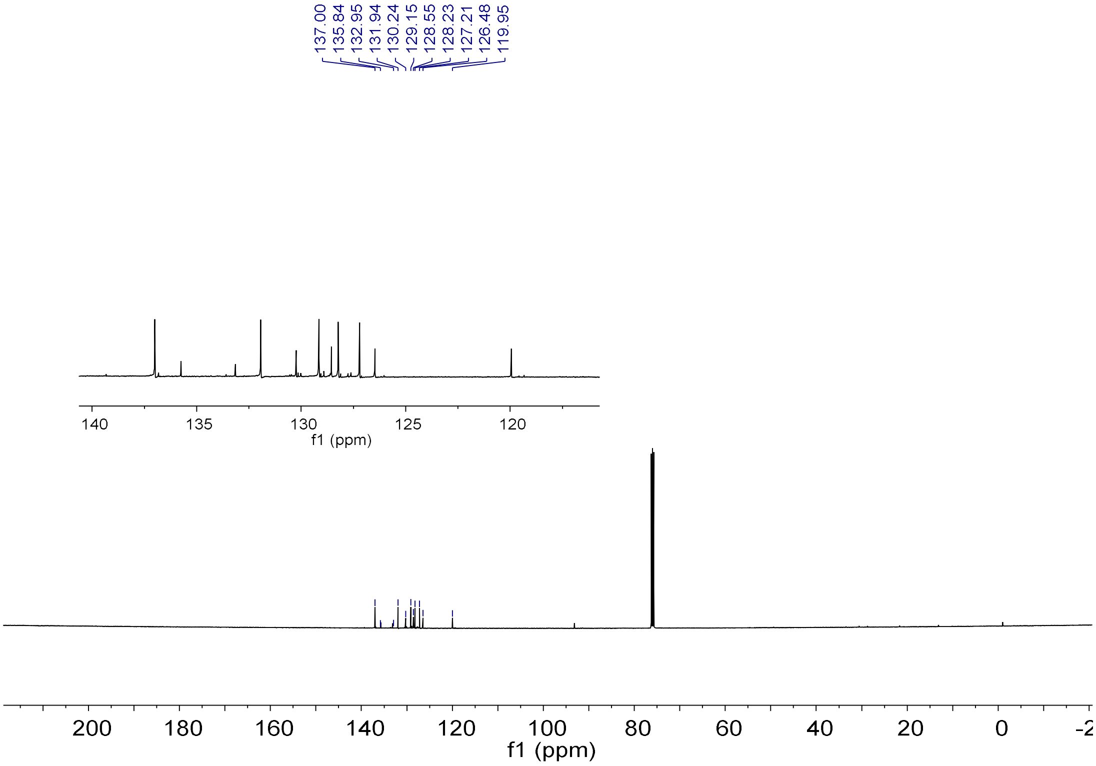

Supplementary Fig. 12 | ^{13}C NMR spectrum of compound **4** in $CDCl_3$ (126 MHz, 298 K).

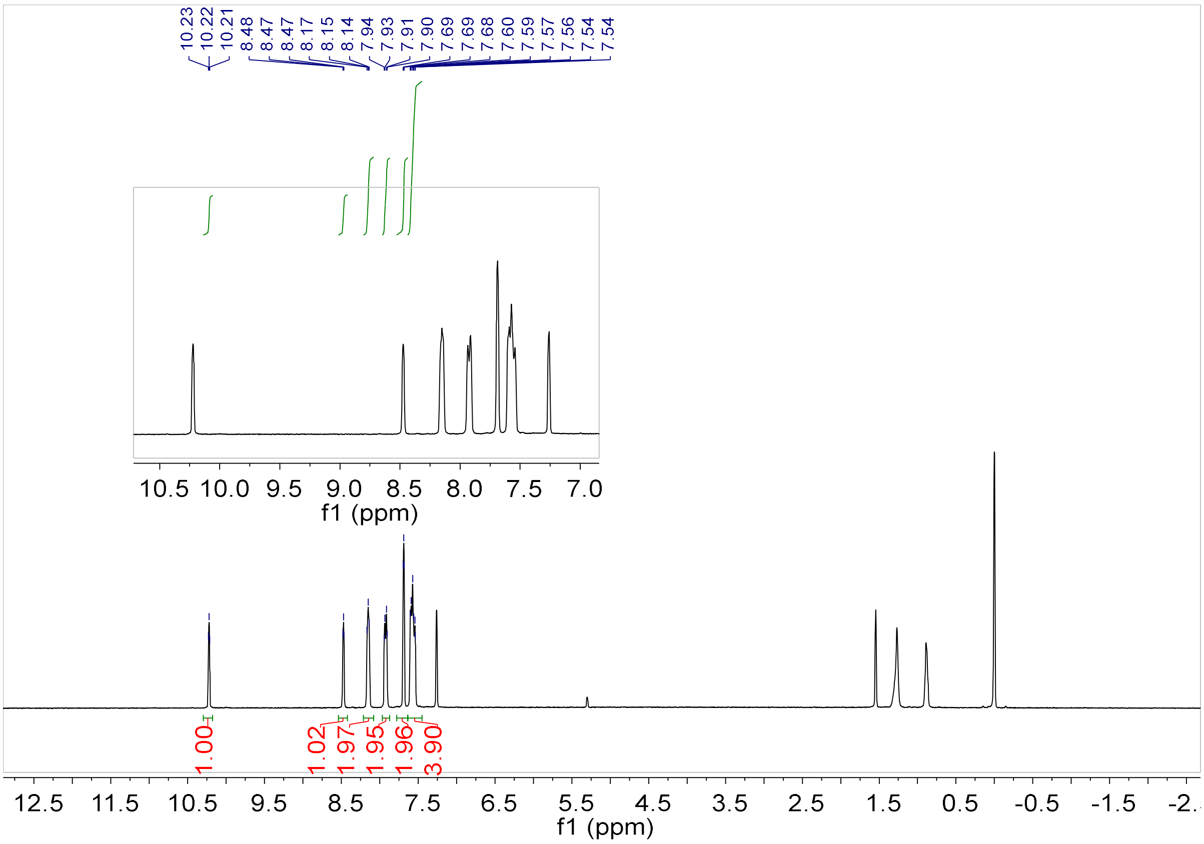

Supplementary Fig. 13 | ^{1}H NMR spectrum of compound **5** in $CDCl_3$ (400 MHz, 298 K).

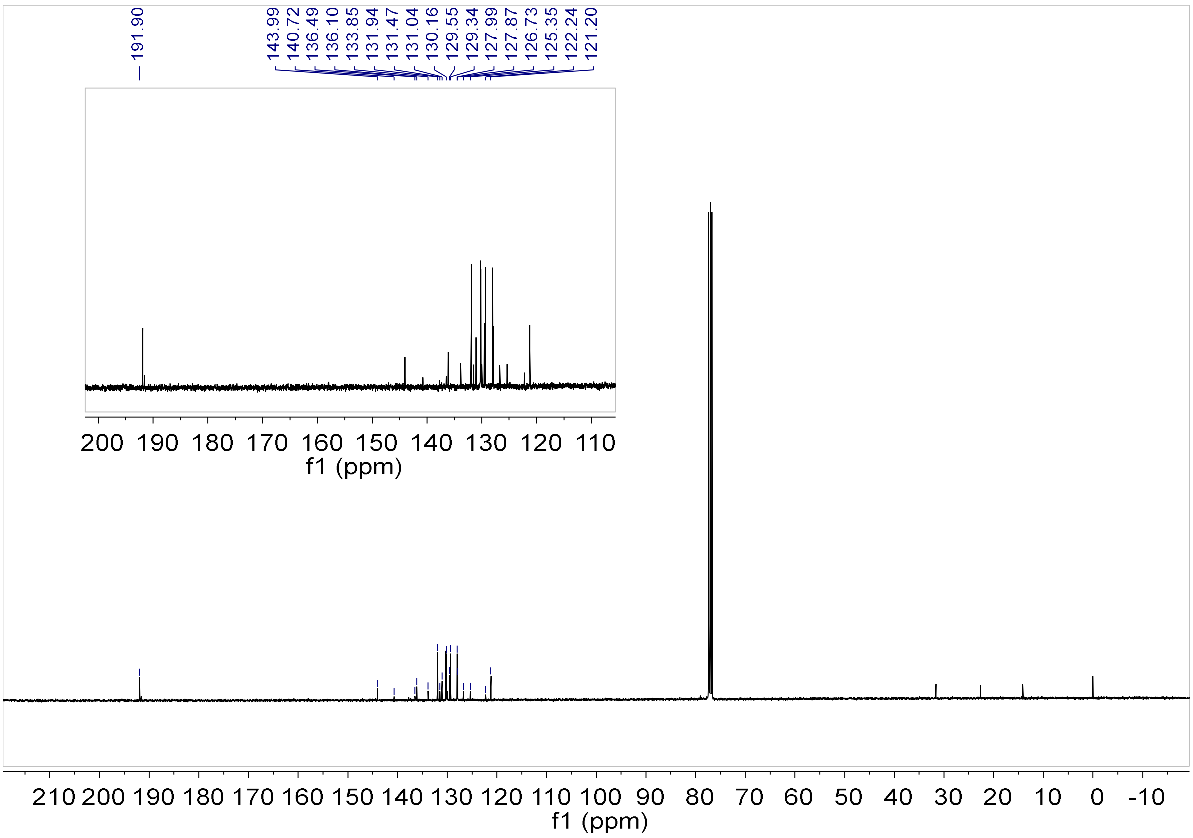

Supplementary Fig. 14 | ^{13}C NMR spectrum of compound **5** in $CDCl_3$ (101 MHz, 298 K).

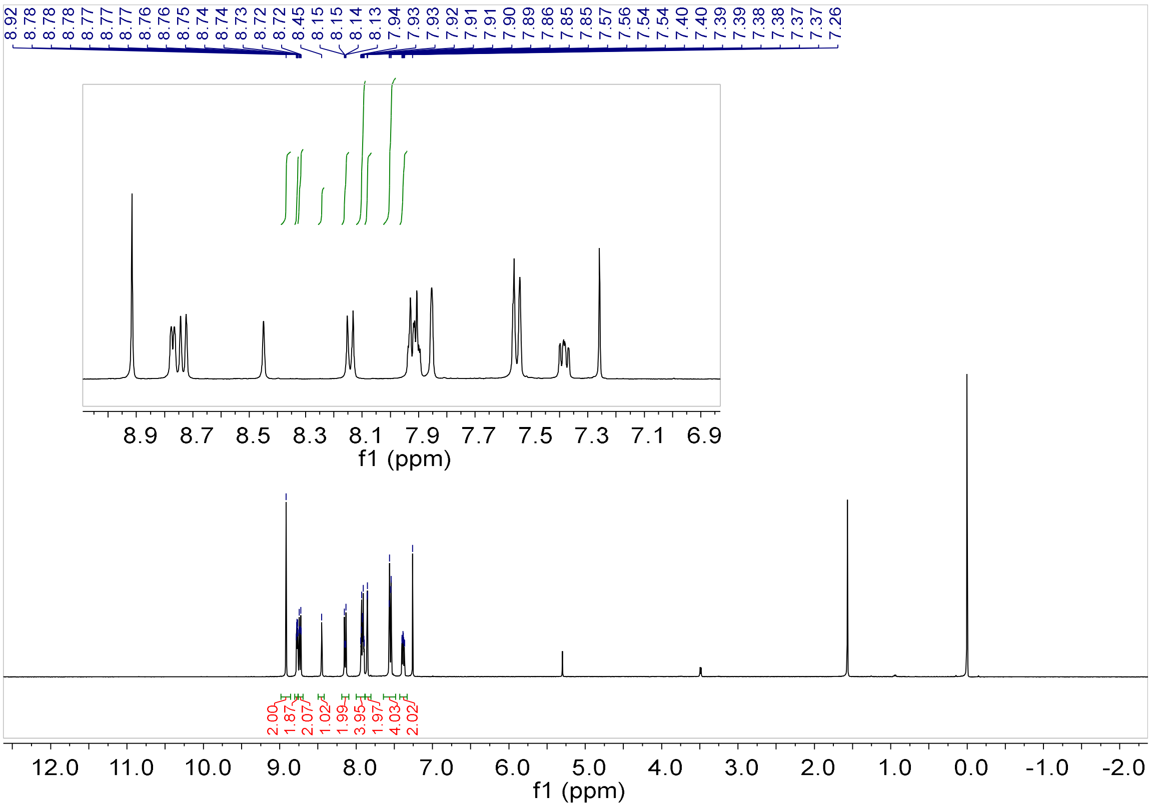

Supplementary Fig. 15 | ^{1}H NMR spectrum of compound **1** in $CDCl_3$ (400 MHz, 298 K).

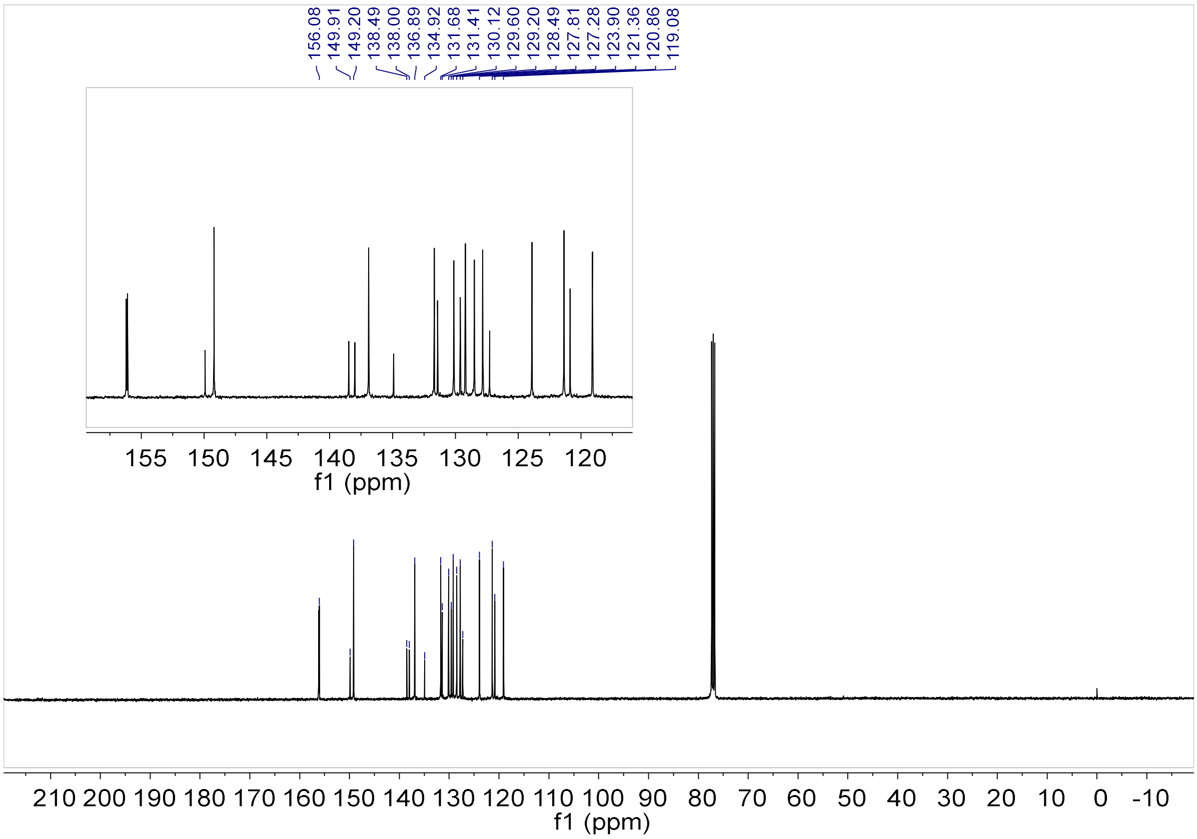

Supplementary Fig. 16 | ^{13}C NMR spectrum of compound **1** in $CDCl_3$ (101 MHz, 298 K).

References

1. Zhang, F.-L., Hong, K., Li, T.-J., Park, H. & Yu, J.-Q. Functionalization of C(sp3)–H bonds using a transient directing group. *Science* **351**, 252–256 (2016).
2. Kinikar, A. *et al.* On-Surface Synthesis of Edge-Extended Zigzag Graphene Nanoribbons. *Advanced Materials* **35**, 2306311 (2023).
3. Wang, M. *et al.* Hexagon Wreaths: Self-Assembly of Discrete Supramolecular Fractal Architectures Using Multitopic Terpyridine Ligands. *J. Am. Chem. Soc.* **136**, 6664–6671 (2014).
